# Comprehensive genomic analysis of a plant growth-promoting rhizobacterium *Pantoea agglomerans* strain P5

**DOI:** 10.1038/s41598-017-15820-9

**Published:** 2017-11-15

**Authors:** Vahid Shariati J., Mohammad Ali Malboobi, Zeinab Tabrizi, Elahe Tavakol, Parviz Owlia, Maryam Safari

**Affiliations:** 1 Plant Molecular Biotechnology Department, National Institute of Genetic Engineering and Biotechnology, Tehran, Iran; 2 R&D Department, Green Biotech Inc., Suite 10, 47 Bu-Ali-Sina St. W, Bistoun Ave, Fatemi Sq, Tehran, Iran; 3 NIGEB Genome Center, National Institute of Genetic Engineering and Biotechnology, Tehran, Iran; 4 Department of Crop Production and Plant Breeding, College of Agriculture Shiraz University, Shiraz, Iran; 5 Molecular Microbiology Research Center, Faculty of Medicine, Shahed University, Tehran, Iran; 6 Energy and Environmental Biotechnology Department, National Institute of Genetic Engineering and Biotechnology, Tehran, Iran

**Keywords:** Comparative genomics, Prokaryote

## Abstract

In this study, we provide a comparative genomic analysis of *Pantoea agglomerans* strain P5 and 10 closely related strains based on phylogenetic analyses. A next-generation shotgun strategy was implemented using the Illumina HiSeq 2500 technology followed by core- and pan-genome analysis. The genome of *P*. *agglomerans* strain P5 contains an assembly size of 5082485 bp with 55.4% G + C content. *P*. *agglomerans* consists of 2981 core and 3159 accessory genes for Coding DNA Sequences (CDSs) based on the pan-genome analysis. Strain P5 can be grouped closely with strains PG734 and 299 R using pan and core genes, respectively. All the predicted and annotated gene sequences were allocated to KEGG pathways. Accordingly,  genes involved in plant growth-promoting (PGP) ability, including phosphate solubilization, IAA and siderophore production, acetoin and 2,3-butanediol synthesis and bacterial secretion, were assigned. This study provides an in-depth view of the PGP characteristics of strain P5, highlighting its potential use in agriculture as a biofertilizer.

## Introduction

The mechanisms and molecular basis of plant growth promoting traits such as phosphate solubilization differ among bacterial species^1^. Phosphate-solubilizing bacteria applied as bio fertilizers assist in the hydrolysis of an extensive range of P compounds that lead to increased growth and yield in plants^2–4^.

The genus *Pantoea* includes several species that are isolated from water, soil, humans, animals and plants as pathogens, symbionts or commensals^5,6^. Distinctive strains of *Pantoea agglomerans* are recognized as plant growth-promoting rhizobacteria (PGPR) and used in the form of soil inoculants as biofertilizers in agriculture. As shown by Malboobi *et al*., the *P*. *agglomerans* strain P5 is one of the important strains that hydrolyze inorganic and organic phosphate compounds effectively^7^ by the conversion of insoluble forms of phosphate into soluble forms through the secretion of organic acids or protons^8^. It is a Gram-negative bacterium that is commercially produced as a phosphate biofertilizer named PhosphoBarvar-2.

Whole-genome shotgun (WGS) sequencing and annotation analysis of the whole-genome data can lead to the identification of genes that are conducive to the beneficial activity of PGPR.

This analysis will provide insight into the molecular mechanisms, functional capabilities and biodiversity of this bacterial species. Therefore, WGS has been recently employed to study the genomes of several PGPRs such as a *Klebsiella* strain^9^, a *Pseudomonas* strain^10^, a *Bacillus* strain^11^, *Paenibacillus polymyxa*^10^, *Enterobacter*, *Erwinia*, *Serratia*, *Alcaligenes*, *Arthrobacter*, *Acinetobacter* and *Flavobacterium*^8,12^. In this study, whole-genome sequencing and annotation of *P*. *agglomerans* strain P5 was performed and compared with the 10 most similar strains through Pan-genome and phylogenetic analyses to determine strain propinquity and to identify target genes and mechanisms that contribute to the beneficial interaction between bacteria and plants that eventually results in plant growth promotion and the improvement of soil fertility^13^. Because of insufficient knowledge regarding to plant growth-promoting function in P5 strain, our comparative genomic study will present a global view of its possible genetic basis.

## Results

### Statistics of *P*. *agglomerans* strain P5 sequences

Next-generation shotgun sequencing using the Illumina HiSeq 2500 platform resulted in approximately 180-fold coverage of the *P*. *agglomerans* strain P5 genome. In total, 10,396,856 raw reads 90 bp in length were subjected to filtering for reads with > 10% Ns and/or 25–35 bases with a low quality average (≤Q20). Finally, 9.919 million clean reads were used for downstream analysis (Tables S1 and S2). *De novo* assembly optimization of the reads resulted in 150 scaffold with total length of 5,082,485 bp using k-mer 21, 33, 55, 73, 89. As a result of final assembly the set of scaffolds (≥500 bp) was defined, consisting of 110 scaffolds with lengths ranging from 902 bp to 280,881 bp (N50 = 112,468 bp), resulting in a total length of 5,070,221 bp (Table S3).

### *P*. *agglomerans* strain P5 genome features and comparative genome analysis

**Figure 1:**
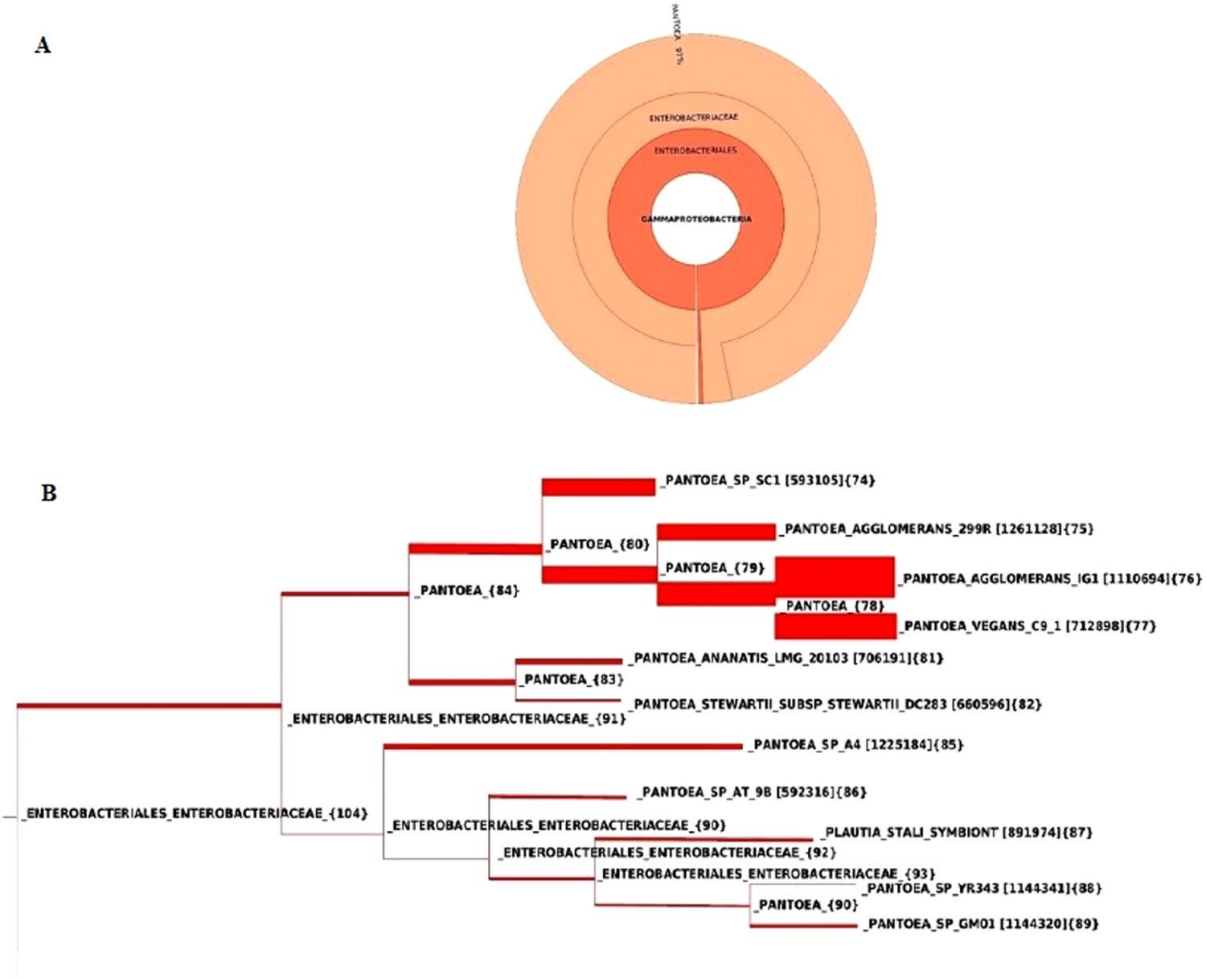
Taxonomic assignment of *P*. *agglomerans* strain P5. The tree was inferred using FastTree from an Hmmalign alignment of P5 reads to 37 concatenated universal single-copy marker genes in Phylosift. The closely related genomes are shown by the red indicator with their GenBank accession numbers provided in parentheses. Here, tree was rooted to *P*. *agglomerans* as the closest species.

**Figure 2:**
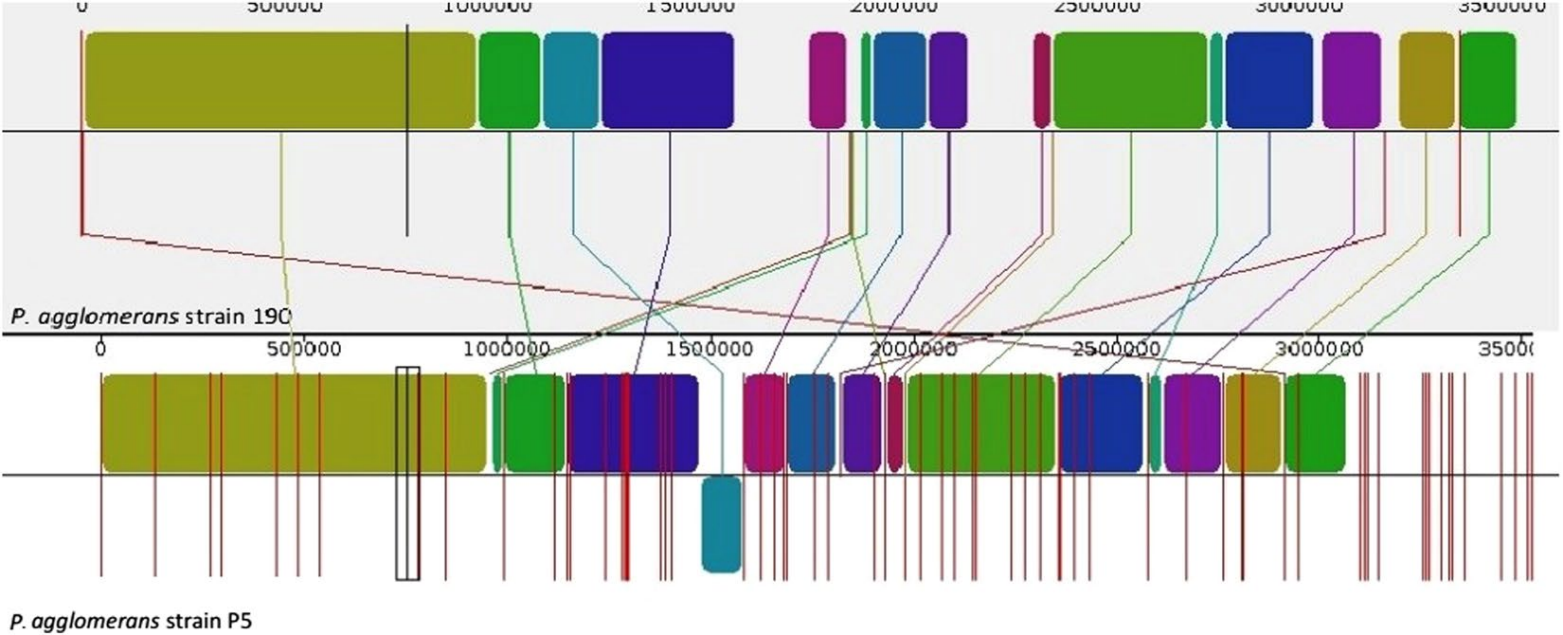
Genome alignment showing syntenic blocks between *P*. *agglomerans* strains 190 (top) and P5 genomes (bottom).

The genome of *P*. *agglomerans* strain P5 has a single circular chromosome of ~5 Mbp with a genomic GC content of 55.4%. A total of 4745 genes were predicted in this genome, encoding 4674 putative coding sequences (CDS). Furthermore, a total of 63 tRNA-coding genes and 7 rRNA genes were predicted in the chromosome sequence. PhyloSift analysis was conducted to reveal phylogenetic models providing high resolution identification of organisms (Fig. 1). Then, to find the best reference sequenced genome, the strain P5 reads were mapped against each of the 10 available relevant genome sequences. Ultimately, *P*. *agglomerans* strain 190 was selected as an appropriate reference genome (Table S1). Using the Gepard dot plot software and progressive Mauve from the Mauve software, we compared the ordered genome assembly of strain P5 with that of strain 190 (GenBank: GCA_000731125.1) as a reference genome. The genome alignment indicated the presence of 20 collinear blocks (Fig. 2), with several regions of inversion and rearrangement. It seems that the chromosomal alignments of these strains are approximately identical, as shown by the existence of large segments of high similarity when most portions of the two chromosomes are mapped onto each other. On the other hand, a region between contigs 4 to 6 in the P5 chromosomal scaffold approximately at 2 Mb of the P5 chromosome displays inversion, revealing different syntenial relationships to the reference sequence.

**Figure 3:**
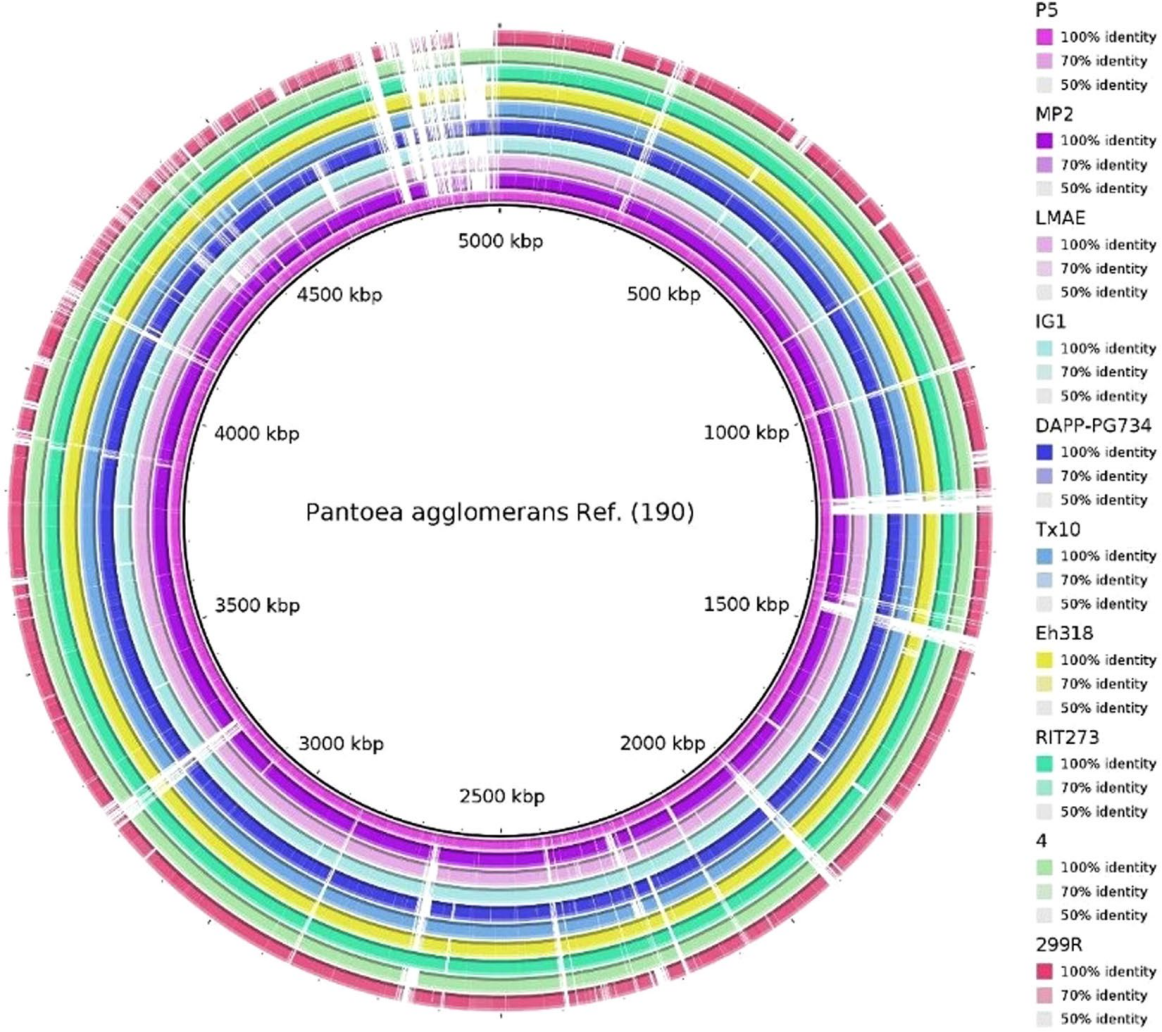
Genome comparisons of P5 strain and other *P*. *agglomerans* strains against reference genome (strain 190) generating by BRIG 0.95, the circular map illustrates the whole genome comparison of strain 190 against the other 10 sequenced *P*. *agglomerans*. The inner cycle (back) represents the complete genome of the reference strain 190 and the shade of each colors show the similarities between each strains with strain 190.

**Figure 4:**
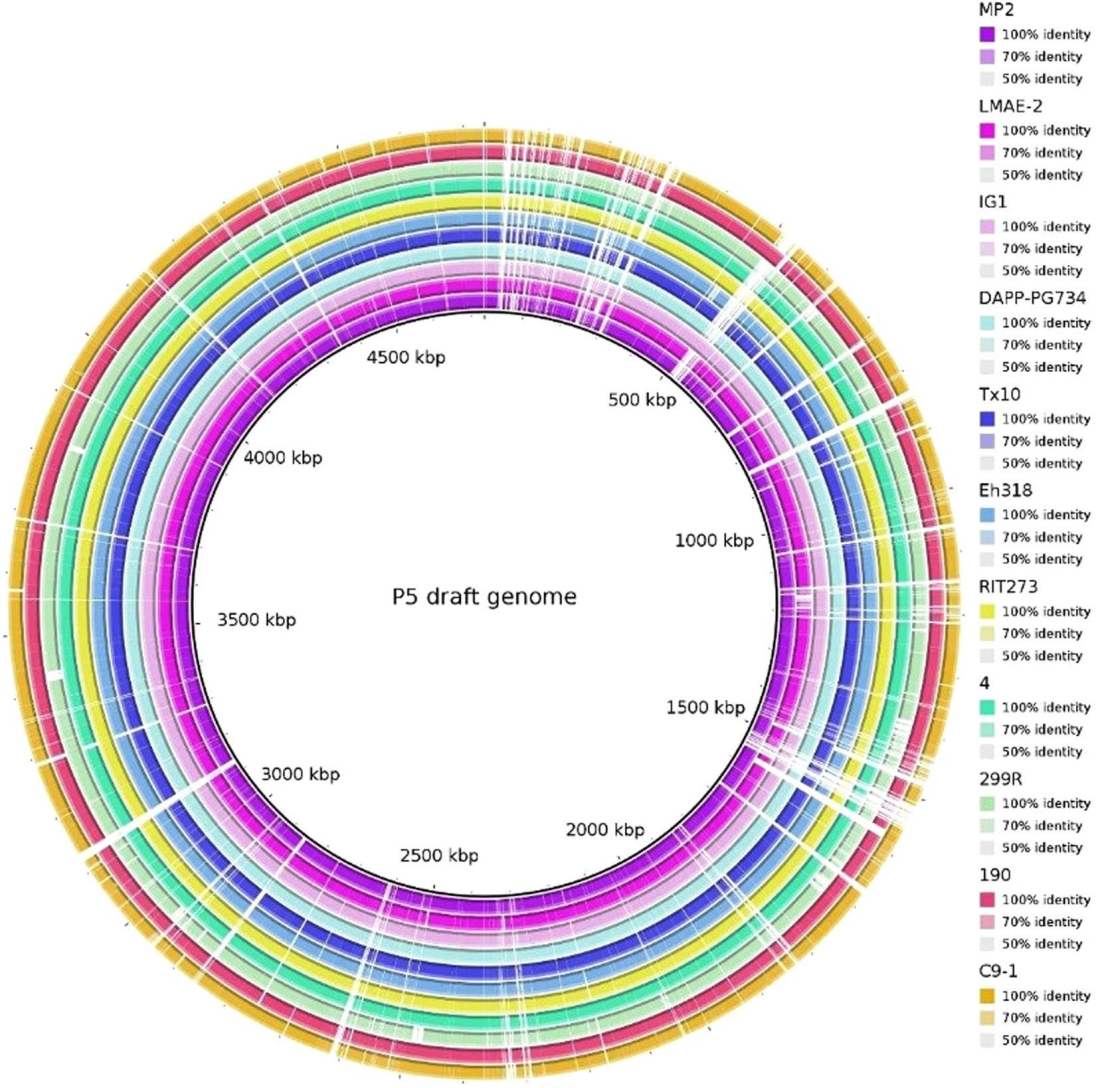
Genome comparisons of other *P*. *agglomerans* strains against drafted P5 genome. The inner cycle (back) represents the complete genome of the reference strain P5 and the shade of each colors show the similarities between each strains with strain P5.

Additional genomic features of *P*. *agglomerans* strain P5 and 10 closely related genomes, such as the sequence similarity, distribution of GC content and the number of genes including tRNA and rRNA, were analyzed (Figs 3 and 4, Table 1). The genome size, total number of genes and total predicted CDS of P5 are similar to but slightly smaller than those of RIT273 and DAPP-PG734, while DAPP-PG734 appears to have a larger genome size of ~5.3 Mb.

**Table 1:** The genomes of *Pantoea agglomerans* strains used for mapping and comparative genome analysis.

Strains	Genome size (Mb)	GC%	No. of rRNA	No. of tRNA	Scaffolds	No. of Genes	No. of Proteins	GenBank Accession no.
P5	5.16726	55.4	7	63	150	4745	4674	GCA_002157425.2
Tx10	4.85699	55.1	48	142	22	4627	4500	GCA_000475055.1
Eh318	5.03584	54.8	28	73	34	4791	4627	GCA_000687245.1
190	5.00257	55.1	24	77	5	4878	4778	GCA_000731125.1
MP2	4.73383	55.2	22	71	16	4352	4214	GCA_000757415.1
IG1	4.82958	55	2	63	18	4443	4341	GCA_000241285.2
299 R	4.58148	54.3	27	63	109	4267	4157	GCA_000330765.1
RIT273	5.36534	55.1	17	76	26	4999	4914	GCA_000627115.1
DAPP-PG734	5.36593	54.7	36	70	195	5107	4937	GCA_000710215.1
4	4.8102	55.1	19	72	12	4370	4260	GCA_000743785.1
LMAE-2	4.98116	55.1	51	79	155	4681	4406	GCA_000814075.1

### *P*. *agglomerans* pan-genome features

A pan-genome for the strain P5 and ten sequenced *P*. *agglomerans* strains was determined by PGAP, comparing of the translated CDS set, followed by clustering of orthologous proteins and the representatives of each orthologous cluster and strain-specific CDS in the total pan-genome. The total pan-genome for the 11 compared *P*. *agglomerans* strains encompasses 6140 CDS. Of these, 2981 (48.5% of total CDS) are core conserved genes across all 11 strain genomes compared. A total of 3159 protein CDS (51.44% of the pan-genome total) constitute the accessory fraction, which are unique to each genome. The smallest numbers of specific genes were encoded by *P*. *agglomerans* strains 4 and MP2, with 85 and 99, respectively.

**Figure 5:**
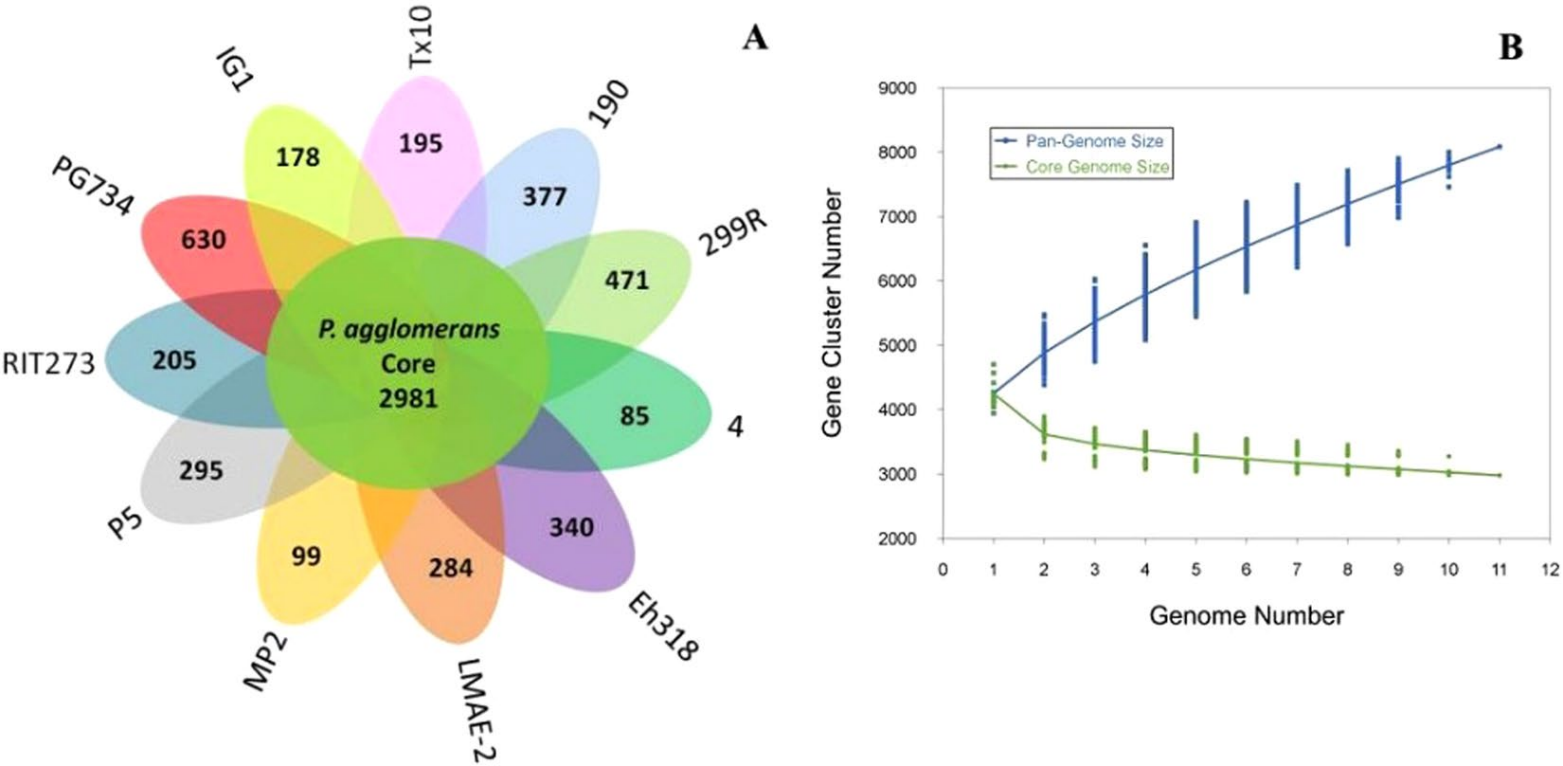
Plot of core/pan-genome and Venn diagram for the core genome and strain- specific CDS of the *P*. *agglomerans* strains. (**A**) The number of unique CDS for each strain of the *P*. *agglomerans* pan-genome. (**B**) Comparisons of n = 1 → 11 genomes to determine the core genome and strain-unique CDS for the eleven sequenced *P*. *agglomerans* strains.

The highest numbers of specific genes belong to *P*. *agglomerans* strains PG734 and 299 R, with 630 and 471, respectively (Fig. 5). The number of unique CDS in the target genome strain P5 was 295 CDS. Several virulence genes have been identified in Gram-negative bacteria, including those related to hypersensitive responses and pathogenicity (hrp), effectors of avirulence (avr) and bacterial toxins, among others. It is well known that the type III secretion system, governed by the *hrp/hrc* gene cluster, promotes bacterial pathogenesis by the secretion of effector proteins directly into the host cell. Hence, to identify the bacterial virulence genes (*hrp/hrc* gene cluster) that may eventually lead to plant disease promotion, we evaluated the set of clusters of orthologous genes shared by *P*. *agglomerans* strain P5 and its 10 closest strains. After studying the core genome and pan-genome, we found the complete *hrc*/*hrp* gene cluster only in strain PG734, whereas we found some *hrp/hrc* genes in the strain P5, 190 and IG1 genomes (Table S4).

As reported in various studies, a bacterial adaptive immune system called the CRISPR-Cas system is acquired by some bacterial species. It has been utilized to improve genome editing technology, ultimately leading to improved viral defense systems. The P5 genome includes genes related to type I CRISPR-Cas systems (Table S5), indicating the presence of a microbial defense system against intrusive elements^14^. Moreover, Pan-genome analysis identified 295 strain specific genes for P5 consisting 55 known genes belonging to phosphotransferase system (PTS), transcription regulators, ABC transporter, transferases and etc. (Table S5).

### Intra-species phylogenetic tree analysis

**Figure 6:**
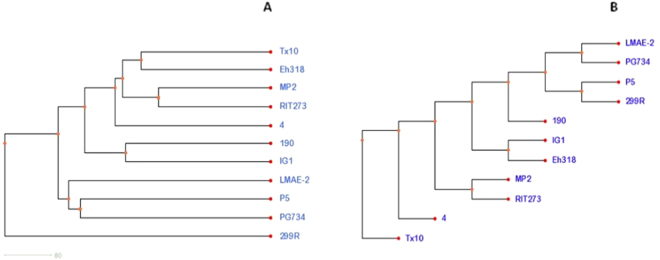
Phylogeny of the *P*. *agglomerans* strains based on the analysis of pan-genomes (**A**) and Single Nucleotide Variants (SNVs) in core genomes (**B**).

The phylogenetic relations of *P*. *agglomerans* strain P5 and 10 other *P*. *agglomerans* strains were demonstrated separately based on the pan-genome and the core genome. As shown in (Fig. 6), analysis based on the pan-genome clustered *P*. *agglomerans* strains P5 and PG734 in the same clade, genetically close to the LMAE-2 genome. This analysis also showed a distant phylogenetic relationship between strain 299 R and the others, whereas 299 R was clustered in the same clade as strain P5 in phylogenetic analysis based on the core genomes. Moreover, strain PG734 and strain LMAE*-2* were clustered together and close to strain P5.

### Genes related to plant growth promotion

The annotation of the *P*. *agglomerans* strain P5 genome identified genes associated with the solubilization of phosphate, indole acetic acid (IAA), siderophores and phytohormone productions that are conducive to plant growth promotion^9,15,16^ It is well known that a large portion of inorganic phosphates become immobilized after application as fertilizer, resulting in the inaccessibility of the phosphate to plants^13^. Therefore, the capability of some bacterial species to solubilize insoluble or insufficiently soluble mineral phosphates by generating acid phosphatases and organic acids, especially gluconic acid, is important^17^.

### Organic acid production and phosphate metabolism

Gluconic acid (GA) is an organic acid that is significantly responsible for the solubilization of mineral phosphates. GA biosynthesis is carried out by glucose-1-dehydrogenase *(gcd)* along with its co-factor pyrrolo-quinolone quinine (PQQ). In addition, gluconic acid dehydrogenase *(gad)* contributes to GA production and its conversion to 2-ketogluconate^18,19^. Accordingly, P5 genome annotation indicated the possession of several genes related to gluconic acid biosynthesis and its co-factor genes, including *pqqBDEF*^19^. Another organic acid identified in the P5 strain that is relevant to the phosphate-solubilizing trait is 2-ketogluconic acid, which is produced by gluconate 2-dehydrogenase alpha/beta chain and 2-keto-D-gluconate reductase. Moreover, the P5 strain of *P*. *agglomerans* was found to produce other organic acids such as lactic, acetic, glycolic, succinate, and citric acid (Table S6).

**Figure 7:**
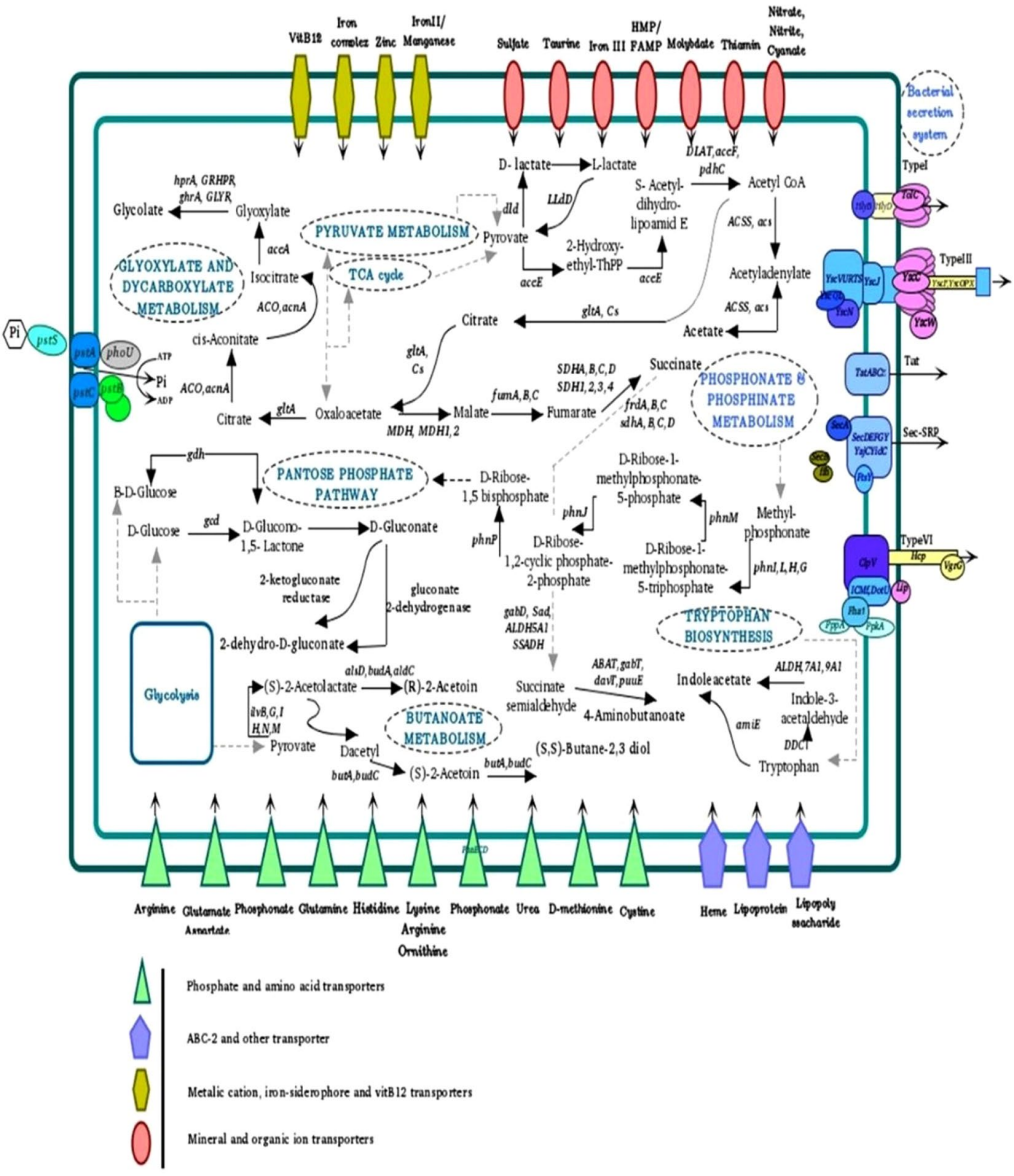
Schematic overview of metabolic pathways of *P*. *agglomerans* strain P5. The portrayed pathways were identified based on the genomic data, which show the strain carries genes consistent with the ability to solubilize phosphate compounds. The genome also includes a complete carbohydrate metabolic pathway involving glycolysis/gluconeogenesis, the tricarboxylic acid (TCA) cycle, pyruvate metabolism and the pentose phosphate pathway (PPP). The latter includes the genes responsible for gluconic acid synthesis from D-glucose, such as *gcd* and *gdh* and the related pathways. Similarly, the synthesis of other organic acids is shown in their specified pathway.

Various studies have indicated that another rich source of soil P is in the form of phosphonate. Phosphonate is an organophosphorus compound that contains a direct carbon-phosphorus (C-P) bond in place of the better known linkage of phosphate esters (C-O-P)^20^. The phosphonate-related *phn* gene cluster is responsible for the release of biologically available phosphate through the bacterial degradation of phosphonates. Our comparative genomic study revealed that strain P5 carries several *phn* genes, including *phnHNIKJGAPBLOEF*, showing the capability of hydrolyzing phosphonate into phosphate and an alkane. Furthermore, the P5 genome encodes *phnA*, which is required for the degradation of phosphonoacetate. These require the route of phosphorus-compound transport across the plasma membrane before utilization. P5 fulfills this task through two high-affinity phosphate transport systems, *PstBACS* (phosphate transporter) and *PhnE2E1DC* (phosphonate transporter) (Table S7). The P5 strain does not carry the necessary genes (*ppd*, *pepM*) for 2-amino-ethylphosphonate and phosphonopyruvate degradation. In addition, it does not carry *phnW*, which is needed for the biosynthesis of phosphonates (Fig. 7).

### Sulfate assimilation

**Figure 8:**
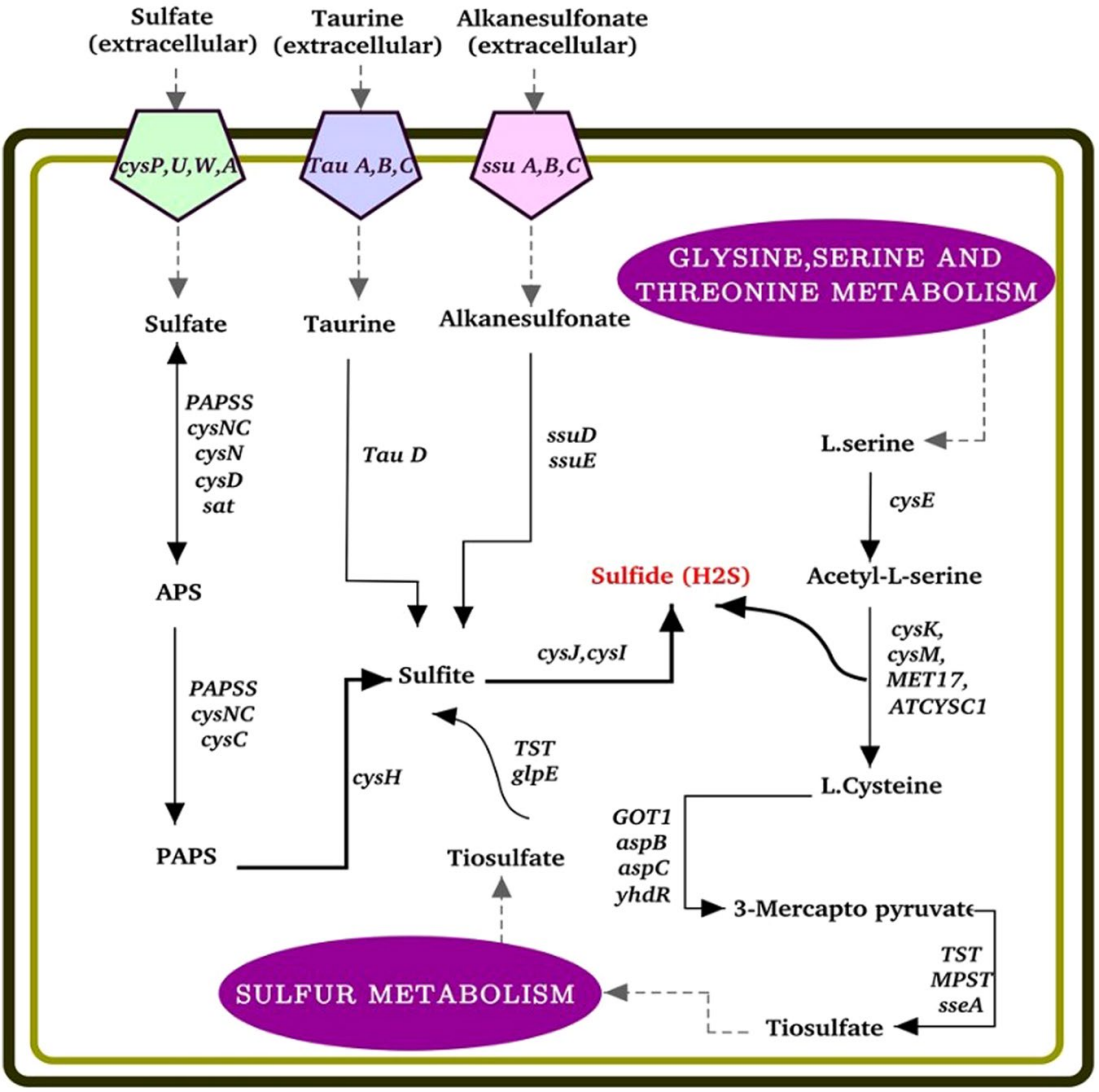
Schematic summary of hydrogen sulfide (H_2_S) production pathways. The figure illustrates the integration of metabolic pathways contributing to the production of sulfite. The metabolism of sulfur in P5 includes the assimilation of inorganic sulfate and the mineralization of organic taurine and sulfonates. The ABC-type transportation system includes a periplasmic binding protein as well as cysPUWA for sulfate, TauABC for taurine and ssuABC for alkanesulfonate transportation. Inorganic sulfite is a common point among four pathways, including 1) sulfate-APS-PAPS, 2) L-cysteine-3-mercaptopyruvate-thiosulfate-sulfite, 3) taurine-sulfite, and 4) alkanesulfonate-sulfite. Sulfite is then reduced to sulfide by cysIJ.

Hydrogen sulfide (H_2_S) has emerged as an important molecule with beneficial effects on phosphate solubilization. H_2_S reacts with ferric phosphate to yield ferrous sulfate and the liberation of phosphate. The sulfur metabolism in strain P5 includes the mineralization of organic sulfonates and taurine or 2-aminoethanesulfonic acid towards the assimilation of inorganic sulfate. The strain P5 genome encodes the set of genes that are responsible for H2S biosynthesis (Table S8, Fig. 8), including the *cysCIJHND*, *PAPSS*, and *sat* genes involved in assimilated sulfate reduction. Thiosulfate is converted to sulfide by the products of the *TST* and *glpE* genes and to homocysteine by cystathionine gamma-synthase (*metB*) enzymes. Furthermore, the strain P5 genome contains the *ssuD* and *ssuE* genes, which are related to the conversion of alkanesulfonate to sulfite, and the *tauD* gene for taurine. On the other hand, the P5 genome also possesses taurine transporter-related proteins including *tauA*, *tauC*, and *tauB*, a sulfonate transport system including *ssuA*, *ssuB*, and *ssuC* and a sulfate transport system including *cysPUWA* (Table S9). In addition, a transcriptional regulator coding gene, *cysB*, that is considered to be the main regulator for sulfur assimilation was identified in the P5 genome.

### Secretion systems

A highly specialized secretion system has developed in bacterial species. The bacterial secretion system plays an important role not only in the response of a bacterium to its environment but also in several physiological processes. We found three potential outer-membrane protein secretion systems, including Types I, III, and VI, as well as two other secretion systems, Tat (twin arginine translocation) and Sec-SRP (general secretory pathway), in the P5 genome (Table S10, Fig. 7).

### Plant growth promotion hormones and volatile compounds

The production and/or activation of phytohormones such as auxin and cytokines or other factors that modulate plant regulatory systems are employed by PGPR to enhance plant growth through the regulation of cellular activities, pattern formation, vegetative and reproductive development and stress resistance^21,22^.

Indole-3-acetic acid (IAA) is the primary auxin endogenously synthesized by PGP bacteria and has an essential effect on plant growth and development processes, such as cell elongation and organogenesis, as well as tropic responses. IAA can be produced from tryptophan via four alternative pathways, including the indole-3-acetamide (IAM), indole-3-pyruvic acid (IPA), tryptamine (TAM) and indole-3-acetaldoxime (IAOX) pathways^23^. Here, P5 carries the amidase gene, which contributes to the indole-3-acetamide conversion to IAA in the IAM pathway. Some genes responsible for IAA biosynthesis, such as *ipdC*, are absent in the P5 genome. These results indicate that the IAM pathway may be the sole route for IAA production in this strain^24^.

Volatile organic compounds (VOCs) including 3-hydroxy-2-butanone (acetoin) and 2,3-butanediol secreted by *P*. *agglomerans* strain P5 may not only promote plant growth by stimulating root formation but also play a role in increasing systemic disease resistance in plant-bacteria interactions^25^. In this study, several genes implicated in acetoin and 2,3-butanediol synthesis, including genes that encode enzymes such as acetolactate synthase, acetolactate decarboxylase, butanediol dehydrogenase and acetoin reductase, were detected^26^. Therefore, the function of these genes could be assigned to growth promotion and systemic disease resistance (Table S11, Fig. 7).

It has been reported that PGPR may produce compounds such as 4-hydroxybenzoate, which act as antibiotics and suppress plant pathogenic microbes, or γ-aminobutyric acid (GABA), which is responsible for pest and disease inhibition. We identified *UbiC*, which is involved in 4-hydroxybenzoate synthesis, and the *gabD* and *gabT* genes, which contribute to GABA synthesis, in the genome of strain P5 (Table S11).

### Siderophore biogenesis

It has been shown that siderophores, high-affinity iron-chelating compounds, are largely produced by bacterial strains associated with plants^27^ and can benefit them by retrieving iron from the environment^21^. Our genomic study showed that P5 is able to synthesize an enterobactin siderophore involving the *EntD*, *EntF*, *EntC*, *EntE*, *EntB* and *EntA* genes. The siderophore is then exported from the cell using *entS* and is responsible for recovering iron by complex formation. Having siderophore receptors, bacteria may heterologously adopt siderophores produced by other organisms as well. *P. agglomerans* strain P5 encodes several genes for siderophore receptors, including *TonB-*dependent outer-membrane receptors and three putative ferric enterobactin receptors (*fepA*) (Table S12).

## Discussion

In this study, we sequenced, analyzed and compared the genome of *P*. *agglomerans* strain P5, which is commercially used as a biofertilizer. The genomic data of strain P5 supported previous observations of its ability to promote plant growth by playing a primary role in solubilization of phosphate^4,7^. Our comparative genomic analysis of *P*. *agglomerans* strain P5 and 10 previously sequenced strains shows high propinquity between these strains, particularly strains DAPP-PG734, LMAE-2 and 190. As confirmation, a phylogenetic tree was constructed based on the pan-genome that revealed *P*. *agglomerans* strains P5 and PG734 to be in the same clade and genetically close to strain LMAE-2 (Fig. 6A). Furthermore, a phylogenetic tree based on the core genome also supports and reveals the low difference in SNV content among these strains (Fig. 6B), which indicates their evolution from a common ancestor.

In addition, our analysis of the core genomes and pan-genomes provided some clues on the specific genes involved in plant disease development and molecular basis of pathogenic capacity among the studied strains of *P*. *agglomerans*.

Many Gram-negative bacteria use type III secretion systems (T3SSs) as injection devices to convey effectors (T3Es) directly into the cytosol of infected host cells and colonize their host organisms^28^. T3SSs are encoded by the *hrc*/*hrp* gene cluster, which is related to hypersensitive responses (HRs) and pathogenicity in susceptible plants. Accordingly, several studies have indicated the contributions of these virulence factors, which are designated pathogenicity-related genes in plant diseases. Hence, we performed a comparison to characterize the genetic differences among all 11 close strains to reveal the genes involved in plant disease development. Here, our analysis of the core genomes and pan-genomes of the 11 strains indicates the presence of the *hrc*/*hrp* gene cluster in only four strains, namely, strains PG734, P5, IG1 and 190 (Table S4). However, the strain PG734 is the only genome containing the complete *hrc*/*hrp* gene cluster and also the only strain with pathogenic activity^29^.

It is noteworthy that the HR-inducing strain PG734, which is commonly isolated inside olive knots, cooperates in the creation and development of olive knot disease. As mentioned by Buonaurio (2015)^29^, strain PG734 is able to convey the *AvrE* effector superfamily, DspA/E, into olive cells. By transferring these effectors, PG734 represses the host defenses and therefore promotes the development of the microbiota inside the olive knot^30,31^. In addition to the role of the *hrpG/hrpX* genes in strain PG734, as a key global coordinator of the expression of multiple different virulence factors such as T3SS effectors or *hrpB*, *hrpD*, and *hrpF*, which are associated with elicitation of the plant hypersensitive response, the significant role of the complete *hrc*/*hrp* gene cluster can be explained by this observation that bacterial *Avr* genes are functionalized only in the presence of a complete set of *hrp* genes^32^. Consequently, without the complete T3SS, P5 is unable to elicit active basal defenses or the hypersensitive response^33,34^. Therefore, P5 can be considered as a safe bio fertilizer without any concern on its possible conversion into pathogen.

It is well known that the chief mechanisms of the solubilization of mineral phosphate are the release of organic acids, protons, and hydroxyl ions and the secretion of enzymes synthesized by soil microorganisms^3,12^. As revealed by Rodriguez *et al*. (2006)^8^, gluconic acid is the most frequent agent produced by phosphate-solubilizing bacteria such as *P*. *agglomerans* (formerly named *Erwinia herbicola*), *Pseudomonas cepacia* and *Burkholderia cepacia*. The analysis of the strain P5 genome revealed that it carries *gcd* and *gad* genes known to be responsible for gluconic acid and 2-keto-D-gluconic acid production, which are involved in solubilizing inorganic mineral phosphates. 2-Ketogluconic acid is also present in *Rhizobium leguminosarum*, *Rhizobium meliloti* and *Bacillus firmus*^8,17^. Gyaneshwar *et al*. (1999) revealed that mineral phosphate solubilization mutants of *Enterobacter asburiae* with deficiency in glucose dehydrogenase (GDH) activity fail to release the phosphate from alkaline soils^35^.

Furthermore, we identified several citrate synthase genes in the strain P5 genome that seem to increase the secretion of organic acids and hence the availability of phosphate to the plant. According to Lopez-Bucio *et al*. (2000), under phosphate limitation, to achieve optimal growth by the overproduction of citrate, a plant can produce more leaf and fruit biomass. This finding indicates the essential role of organic acid synthesis genes in P uptake in plants^36^. Similarly, genes related to the production of other organic acids including glycolic acid, lactic acid, citrate and succinate were detected, which have also been identified among phosphate solubilizers^4,37^.

Consequently, it could be assumed that any gene involved in organic acid synthesis might have a profound effect on this ability. Phosphate-specific transporter systems have already been reported in *E*. *coli*, *B*. *subtilis*, *P*. *polymyxa* and *Klebsiella*^9,13,38^. Here, we showed that strain P5 also carries the *pst* operon, including *pstS*, *pstC*, *pstA* and *pstB*. *PstS* is a binding protein, *PstC* and *PstA* are two integral inner membrane proteins, and *PstB* is an ATP-binding protein^39^. Therefore, strain P5 takes up inorganic phosphate partially through a phosphate transport system.

Under phosphate starvation conditions, when readily soluble forms of phosphate compounds are not present, microorganisms have evolved to utilize additional organophosphorus sources, including phosphonates. The enzymes of phosphonate catabolism are conserved in bacteria and encoded by orthologous genes. The genes for phosphonate uptake and degradation in *E*. *coli* were shown to be fourteen in number (*phnCDEFGHIJKLMNOP*)^40^. Similarly, the genomic analysis of strain P5 revealed the presence of *phn* genes *(phnHNIKJGAPBLOEF)* responsible for solubilizing organic phosphate. *PhnC*, *PhnD* and *PhnE* constitute a phosphonate transporter, while *PhnG*, *PhnH*, *PhnI*, *PhnJ*, *PhnK* and *PhnL* are required to degrade phosphonate into phosphate and an alkane, and *PhnF* and *PhnO* play regulatory roles^20^. Moreover, the strain P5 genome encodes *phnA*, which is required for the degradation of phosphonoacetate^40^. The production of H_2_S is the other mechanism of phosphate solubilization. H_2_S then reacts with ferric phosphate to yield ferrous sulfate with the concomitant release of phosphate. Here, we showed that the strain P5 genome encodes the *TST*, *cys*j, and *cys*I genes, which contribute to producing H_2_S from thiosulfate, as well as cysteine synthases that are reported to produce H_2_S in *E*. *coli*^41^.

Consistent with the PGPR properties reported in other studies^9,11,13,24^, we detected some genes in the strain P5 genome that are associated with IAA production, siderophore, acetoin, and 2,3-butanediol synthesis and the bacterial secretion system. As shown in *Enterobacter* strain 638, increased levels of phytohormones including IAA, acetoin and 2,3-butanediol stimulate root development, resulting in better access to nutrients and water, which in turn increases plant growth^24^. Moreover, the production of acetoin and 2, 3-butanediol by PGPR has been reported to increase systemic acquired resistance (SAR) and drought tolerance. Whereas most reports of PGPR-mediated SAR include free-living rhizobacterial strains, there are some reports indicating SAR activity in endophytic bacteria. For instance, SAR was activated by *P*. *fluorescens EP1* against red rot caused by *Colletotrichum falcatum* on sugarcane, by *Burkholderia phytofirmans PsJN* against *Botrytis cinerea* on grapevine and by *Verticllium dahliae* on tomato. In addition to its direct plant growth-promoting abilities, strain P5 carries many genes that can indirectly stimulate plant health by suppressing pathogens. It is able to protect its host against bacterial and fungal infections through efficient competition for essential nutrients such as iron and via producing antimicrobial compounds such as 4-hydroxybenzoate and GABA. Identification of the genes related to the production of antimicrobial compounds, particularly those that stimulate antibiotic production and activity, suggests the biocontrol capability of strain P5 in addition to its role as a biofertilizer.

## Conclusion

Analysis of the *P*. *agglomerans* strain P5 genome confirmed its abilities as a PGPR through revealing several potential genes involved in plant growth promotion, such as phosphate solubilization, siderophore biogenesis, H2S synthesis, and the biosynthesis of hormones and volatile compounds including indole-3-acetic acid, acetoin and 2,3-butanediol. Moreover, the P5 genome contains a set of genes that act as antibiotics and suppression agents toward plant pathogenic microbes. Furthermore, the whole-genome comparison of *P*. *agglomerans* strain P5 with other 10 closely related *Pantoea* strains showed a total of 2981 (48.5% of total CDS) and 3159 (51.44% of the pan-genome total) CDS as the core conserved genes and the accessory fraction, respectively. This work aims to present a comprehensive view of strain P5, which will surely help to provide additional insight into unraveling the complex biological mechanisms that P5 and other similar organisms use to promote plant growth. However, the detailed elucidation of how these genes are regulated and how they function requires further research.

## Materials and Methods

### Growth conditions and genomic DNA preparation

*Pantoea agglomerans* strain P5, kindly provided by Green Biotech Inc., was initially grown at room temperature on Sperber medium composed of 10 g of glucose, 0.5 g of yeast extract, 0.1 g of CaCl_2_, 0.25 g of MgSO_4_·7H_2_O supplemented with 2.5 g of Ca_3_(PO_4_)_2_ and 15 g of agar per liter. After 16 h of incubation, the colonies were picked and sub-cultured as above until a pure culture was obtained. The total genomic DNA of *P*. *agglomerans* strain P5 was extracted by using the QIAamp DNA Mini Kit (Qiagen, CA). After extraction, the quality of the DNA was measured using a NanoDrop™ spectrophotometer (Thermo Scientific NanoDrop 2000c).

### Genome sequencing and assembly

The whole genome was sequenced using the Illumina HiSeq 2500 platform (90 bp paired-end reads). Libraries were generated from 1 mg of genomic DNA using the Nextera DNA Library Preparation Kit according to the manufacturer’s protocol. The quality control of raw sequences was performed by FastQC v0.11.3 followed by adapter and quality trimming via Trimmomatic 0.32. To identify the bacterial species and the most closely related organism with an available reference genome, phylogenetic analysis was conducted using the PhyloSift software (http://phylosift.wordpress.com), As confirmation, we used Bowtie2 v2.3.0 aligner to map sequencing reads to all the available reference genomes for the identified species. Additionally, *de novo* assembly was performed using SPades v3.9.0, with different k-mer sizes and combinations. The quality of each assembly was evaluated using QUAST v3.2. Finally, the k-mer combination (21, 33, 55, 73, 89) was selected as the final and optimum parameter, resulting in 150 scaffolds that were ordered by ABACAS using the closest genome reference (strain 190).

### Annotation

The gene detection and genome annotation of the ordered assembled genome were performed using the RAST (http://rast.nmpdr.org/) server as well as BlastKOALA v2.1 (http://www.kegg.jp/blastkoala/). Prodigal v2.6.2 and Prokka v1.10 were used to predict the bacterial protein-coding sequences of the complete genome sequence (150 scaffold). General genome features such as rRNA and tRNA were filtered and reported using the provided annotations by an in-house script. Subsequently, the annotated genes were investigated to identify their involvement in plant growth-promoting pathways.

### Gene network/pathway analysis

The predicted and annotated gene sequences were analyzed to determine the existence of certain pathways. Then, the corresponding pathway was completely identified by manual inspection of the assigned gene functions based on comparison to the KEGG (Kyoto Encyclopedia of Genes and Genomes) pathways. To illustrate known biological processes, the pathways were graphically represented using PathVisio 3.2.0, (http://www.pathvisio.org/).

### Whole-genome alignment and pan-genome analysis

To study genome rearrangements in *P*. *agglomerans* strain P5 and related bacteria, we used the progressive Mauve program in Mauve v2.3.0 to construct and visualize the multiple genome alignments of the complete genome sequences of *P*. *agglomerans* strain 190 (GCA_000731125.1) and the P5 draft genome. To identify the core and strain- specific genes, a pan-genome analysis of *P*. *agglomerans* strains including P5 and its 10 close strains was carried out by PGAP. Similarly, the phylogenetic tree was constructed using PGAP, based on the SNPs for the core genome and the pan-genome.

### Comparative genome analysis

We compared the genome of *P*. *agglomerans* strain P5 to 10 closely related genomes using the NCBI databases. Accordingly, several genomic features, including the genome size, the number of genes, the number of tRNAs and rRNAs, and the GC contents of the participating strains were compared. Moreover, pairwise genome alignment of all 11 strains was performed and represented by BRIG 0.95.

### Data availability

All data generated during this study are included in this published article and its supplementary information files. Furthermore, extra information about bacterial assembly is available in https://www.ncbi.nlm.nih.gov with Bio Project code: PRJNA386632.

## Electronic supplementary material

Supplementary file
