# Author Correction: Comprehensive genomic analysis of a plant growth-promoting rhizobacterium *Pantoea agglomerans* strain P5

**DOI:** 10.1038/s41598-020-69169-7

**Published:** 2020-07-21

**Authors:** Vahid Shariati J, Mohammad Ali Malboobi, Zeinab Tabrizi, Elahe Tavakol, Parviz Owlia, Maryam Safari

**Affiliations:** 1 Plant Molecular Biotechnology Department, National Institute of Genetic Engineering and Biotechnology, Tehran, Iran; 2 R&D Department, Green Biotech Inc., Suite 10, 47 Bu-Ali-Sina St. W, Bistoun Ave, Fatemi Sq, Tehran, Iran; 3 NIGEB Genome Center, National Institute of Genetic Engineering and Biotechnology, Tehran, Iran; 4 Department of Crop Production and Plant Breeding, College of Agriculture Shiraz University, Shiraz, Iran; 5 Molecular Microbiology Research Center, Faculty of Medicine, Shahed University, Tehran, Iran; 6 Energy and Environmental Biotechnology Department, National Institute of Genetic Engineering and Biotechnology, Tehran, Iran

Correction to: *Scientific Reports* 10.1038/s41598-017-15820-9, published online 15 November 2017

The original version of this Article contained a typographical error in the spelling of the author Parviz Owlia, which was incorrectly given as Parviz Owilia. This has now been corrected in the PDF and HTML versions of the Article, and in the accompanying Supplementary Information file.

