# Supplementary material for: Comprehensive genomic analysis of a plant growth-promoting rhizobacterium Pantoea agglomerans strain P5
Source: Sci Rep. 2017 Nov 15;7:15610. doi: 10.1038/s41598-017-15820-9 (PMC5688152; doi:10.1038/s41598-017-15820-9)
Supplement: Supplementary file 1 — Supplementary file [file 41598_2017_15820_MOESM1_ESM.pdf]

Scientific Reports **Supporting Information**

Comprehensive genomic analysis of plant growth-promoting rhizobacterium Pantoea agglomerans strain P5

Vahid Shariati J.[1,3], Mohammad Ali Malboobi[1,2], Zeinab Tabrizi[1,3], Elahe Tavakol[4], Parviz Owlia[5], Maryam Safari[6]

1. Plant Molecular Biotechnology Department, National Institute of Genetic Engineering and Biotechnology, Tehran, Iran
2. R&D Department, Green Biotech Inc., Suite 10, 47 Bu-Ali-Sina St. W, Bistoun Ave., Fatemi Sq., Tehran, Iran
3. NIGEB Genome Center, National Institute of Genetic Engineering and Biotechnology, Tehran, Iran
4. Department of Crop Production and Plant Breeding, College of Agriculture Shiraz University, Shiraz, Iran
5. Molecular Microbiology Research Center, Faculty of Medicine, Shahed University, Tehran, Iran
6. Energy and Environmental Biotechnology Department, National Institute of Genetic Engineering and Biotechnology, Tehran, Iran

Corresponding author:

Mohammad Ali Malboobi, Ph.D.
Email: malboobi@nigeb.ac.ir

Table S1 Percentage of reads mapped on the best reference sequenced genome of *P. agglomerans* strain 190

Sample	No. of reads	Total no. of bases	Percentage of reads passed filtering	Average length of reads (bases)	Longest read (bases)	Percentage of mapped read
P. agglomerans sp. P5 F	4,959,985	415,929,859	100.00%	83.9	90	90.70%
P. agglomerans sp. P5 R	4,959,937	381,126,821	100.00%	76.8	90	90.30%
Total	9,919,922	797,056,680	100.00%	80.3	90	90.50%

Table S2 Raw and clean sequences statistics

Type	Raw sequence	Clean sequence
Reads (M)	10,396	9.919
Bases (Mb)	935,717	797.056
mLen	90	80.3
pLen	90	90
Q20	96.28%	98.28%
GC%	55.40%	55%

Note: 1. Read (M): Total Number of Reads in million. 2. Bases(Mb): Total Number of Bases in Mb. 3. mLen: Mean Lenth of Reads. 4. pLen: Peak Length of Reads. 5. Q20: Percent of base number calls with quality value of 20 or higher (Q20+) (%). 6. GC%: The percentage of GC content.

Table S3 QUAST report.

	P5 Scaffolds	P5 Scaffolds broken
# contigs (>= 0 bp)	150	145
# Contigs (> = 1000 bp)	102	127
# Contigs (> = 5000 bp)	83	104
# Contigs (> = 10000 bp)	69	91
# Contigs (> = 25000 bp)	55	68
# Contigs (> = 50000 bp)	33	34
Total length (> = 0 bp)	5082485	5065933
Total length (> = 1000 bp)	5063927	5056264
Total length (> = 5000 bp)	5018370	4995683
Total length (> = 10000 bp)	4927637	4906128
Total length (> = 25000 bp)	4701771	4520697
Total length (> = 50000 bp)	3906692	3268103
# Contigs	110	145
Largest contig	280881	280881
Total length	5070221	5065933
GC (%)	54.88	54.88
N50	112468	77519
N75	52905	38775
L50	16	22
L75	32	47
# N's per 100 kbp	84.93	0.36

All statistics are based on contigs of size > = 500 bp, unless otherwise noted (e.g., # contigs (> = 0 bp) and total length (> = 0 bp) include all contigs).

Table S4 Hrp/Hrc Gene Cluster

Trait	COG ID	Gene annotation	KO/Gene ID	KEGG GENE NAME	Strain			
					P5	190	PG734	IG1
**Hrp/Hrc Gene Cluster**	-	Type III secretion protein W	K04058	yscW, sctW			*	
	-	Putative HrpW chaperone	-	-			*	
	-	Type III effector HopZ3	K08598	avrA			*	
	-	Type III secretion inner membrane channel protein (LcrD,HrcV,EscV,SsaV)	K03230	-	*	*	*	*
	-	Type III secretion protein HrpT	-	HrpT			*	
	COG1766	Type III secretion bridge between inner and outermembrane lipoprotein	K03222	(YscJ,HrcJ,EscJ, PscJ)	*	*	*	*
	-	Type III secretion protein F	K03221	yscF, sctF			*	
	-	Type III secretion protein G	-	HrpG			*	
	COG1684	Type III secretion inner membrane protein (YscT, HrcT, SpaR, EscT, EpaR1, homologous to flagellar export components)	K03228	YscT,HrcT,SpaR,EscT,EpaR1	*	*	*	*
	-	Type III secretion protein HrpB(Pto)	-	YscI/HrpB			*	
	-	Hrp pili protein hrpA (TTSS pilin hrpA1)	-	hrpA1			*	
	-	Type III secretion protein HrpJ	K04058	yscW, sctW			*	
	COG1987	Type III secretion inner membrane protein (YscS,homologous to flagellar export components)	K03227	YscS	*	*	*	*
	-	Type III secretion protein HrpQ	K03220.	yscD, sctD			*	
	-	Type III secretion spans bacterial envelope protein	K04056	yscO, sctO			*	
	-	Type III secretion protein P	-	HrpP			*	
	COG1377	Type III secretion inner membrane protein (YscU,SpaS,EscU,HrcU,SsaU, homologous to flagellar export components)	K03229	yscU, sctU, hrcU	*	*	*	*
	COG1317	Type III secretion cytoplasmic protein	K03223	yscL, sctL			*	
	-	Type III secretion protein HrpD	-	HrpD			*	
	-	Type III secretion outermembrane pore forming protein (YscC,MxiD,HrcC, InvG)	K03219	yscC, sctC	*	*	*	*
	COG1886	Type III secretion protein HrcQb	K03225	yscQ, sctQ, hrcQ			*	
	COG1338	Type III secretion inner membrane protein (YscR,SpaR,HrcR,EscR,homologous to flagellar export components)	K03226	YscR,SpaR,HrcR,EscR	*	*	*	*

* represent the existence of listed genes in each strains based on core-pan genome data.

Table S5 P5 strain-specific genes

Trait	COG ID	Gene annotation	KO/Gene ID	KEGG GENE NAME	Strains
CRISPR-Cas system	COG1203	CRISPR-associated endonuclease/helicase Cas3	K07012	cas3	P5
CRISPR-Cas system	-	CRISPR system Cascade subunit CasA	K19123	casA, cse1	P5
CRISPR-Cas system	-	CRISPR system Cascade subunit CasB	K19046	casB, cse2	P5
CRISPR-Cas system	-	CRISPR system Cascade subunit CasE	K19126	casE, cse3	P5
CRISPR-Cas system	-	CRISPR system Cascade subunit CasC	K19124	casC, cse4	P5
CRISPR-Cas system	-	CRISPR system Cascade subunit CasD	K19125	casD, cse5	P5
ABC transporter	COG1137	Putative ATP-binding component of a transport system	K06861	yhbG	P5
ABC transporter	COG1134	Teichoic acid transport system ATP-binding protein	K09693	tagH	P5
ABC transporter	-	Corrinoid ABC transporter ATPase	-	btuD	P5
Phosphotransferase system (PTS)	COG1447	PTS system, cellobiose -specific IIA component	K02759	PTS-Cel-EIIA, celC, chbA	P5
Phosphotransferase system (PTS)	COG1440	PTS system, cellobiose-specific IIB component	K02760	PTS-Cel-EIIB, celA, chbB	P5
Phosphotransferase system (PTS)	COG1440	PTS system, cellobiose-specific IIC component	K02761	PTS-Cel-EIIB, celA, chbB	P5
Transcription factors	COG3711	Transcriptional antiterminator of lichenan operon, BglG family	K03491	licR	P5
Transcription factors	COG0583	HTH-type transcriptional regulator	K16135.	dmlR	P5
Transcription regulation	-	Transcriptional regulatory protein, C terminal domain	-	HMPREF1248_0490	P5
Transcription regulation	-	Response regulator of zinc sigma-54-dependent two-component system	-	LTSEMON_5829	P5
Transferase activity	-	Puative glycosyltransferase	-	YkoT	P5
Transferase activity	COG1835.	O-acetyltransferase	-	OatA	P5
Transferase activity	COG2225	Malate synthase A	K01638	aceB	P5
Transferase activity	-	Acyltransferase 3	-	-	P5
Methyl-transferase	-	Type II DNA modification methyltransferase	K01854	-	P5
Methyl-transferase	-	2-O-methyltransferase NoeI	K21402	noeI	P5
Isomerases	COG0562	UDP-galactopyranose mutase;	-	glf	P5
Isomerases	COG1047	FKBP-type peptidyl-prolyl cis-trans isomerase	K03775	slyD	P5
-	-	Propanediol utilization polyhedral body protein	-	pduA	P5
-	-	Ethidium bromide-methyl viologen resistance protein	-	EmrE	P5
-	-	Endo-1,4-beta-xylanase A precursor	K13465	EIX	P5
-	-	Anti-anti-sigma factor RsbV	K04749	-	P5
RNA polymerase-associated proteins	COG1366	Anti-sigma B factor antagonist	K04749	rsbV	P5

-	-	Protein Gp49	-	-	P5
-	-	Peptidyl-Asp metalloendopeptidase;	-	-	P5
**Endonuclease activity**	-	Putative type II restriction endonuclease	-	cglIR	P5
-	-	LSU ribosomal protein L4p (L1e)	K03010	-	P5
**Nucleotidyl transferases**	COG3344	Retron-type RNA-directed DNA polymerase	K00986	-	P5
**Acyl-transferase**	COG1835	Putative lipopolysaccharide modification acyltransferase	-	Pfl01_2610	P5
**Phosphorelay sensor kinase activity**	-	Sensory box histidine kinase	-	PP_3545	P5
**Chaperone**	COG3121	Chaperone protein	-	papD	P5
**Serine-type endopeptidase activity**	COG1404	Calcium-dependent protease	-	prcA	P5
-	COG1474	Cell division control protein 6	K02213	CDC6	P5
**Alcohol dehydrogenase activity**	COG1062	S-(hydroxymethyl)glutathione dehydrogenase / alcohol dehydrogenase	K00121.	frmA, ADH5, adhC	P5
**Stress response**	-	Stress response protein	-	YsnF	P5
-	COG1974	DNA polymerase V subunit	K03503	DPO5D, umuD	P5
**DNA binding**	COG0732	Type-1 restriction enzyme EcoKI specificity protein	K01154	hsdS	P5
-	-	Anti-RssB factor	-	AN688_0223255	P5
**Hydrolases**	COG0363	Glucosamine-6-phosphate deaminase	K02564	nagB, GNPDA	P5
-	COG3203	outer membrane pore protein F	K09476	ompF	P5
-	COG2723	6-phospho-beta-glucosidase	K01223	bglA	P5
-	-	Phosphoglycerate mutase family	K01837	BPGM	P5
**Hydrolase activity**	-	Organophosphate pesticide hydrolase	-	mpd	P5
**Lyases**	COG0346	Lactoylglutathione lyase;	K01759	GLO1, gloA	P5
-	COG2801	Putative transposase	K07497	-	P5
-	COG0236	Acyl carrier protein	K02078.	acpP	P5
-	COG0287	Prephenate dehydrogenase	K00210	-	P5
**Putative transposase**	COG2801	IS2 transposase TnpB	K07497	insD1	P5
**Oxidoreductase**	COG2303	Gluconate 2-dehydrogenase flavoprotein<br>EC 1.1.99.3	K06151<br>K06152	E1.1.99.3A<br>E1.1.99.3G	P5
**Nucleic acid binding**	-	Integrase core domain protein	-	TTE1156	P5

Table S6 Gene annotations of *P agglomerans* strain P5 genome related to its phosphate solubilization ability

	Trait	COG ID	Gene annotation	KO/Gene ID	KEGG GENE NAME	EC Number
Pentose phosphate pathway	**Gluconic acid**	COG4993	quinoprotein glucose dehydrogenase	K00117	gcd	EC 1.1.5.2
		COG1028	glucose 1-dehydrogenase	K00034	gdh	EC 1.1.1.47
		COG5424	pyrroloquinoline-quinone synthase C	K06137	PqqC	EC 1. 3.3.11
		COG1025	coenzyme PQQ synthesis protein F	-	pqqF	-
		COG1235	Coenzyme PQQ synthesis protein B	K06136	pqqB	-
		-	Coenzyme PQQ synthesis protein D	K06138	pqqD	-
		COG0535	Coenzyme PQQ synthesis protein E	K06139	pqqE	-
	**2-Keto-D-gluconic acid**	COG2303	gluconate 2-dehydrogenase alpha chain	K06151	-	EC 1.1.99.3A
		-	gluconate 2-dehydrogenase gamma chain	K06152	-	EC 1.1.99.3G
		COG1052	- 2-keto-D-gluconate reductase<br>- gluconate 2-dehydrogenase	K00090	-	EC 1.1.1.215
Glyoxylate and dicarboxylate metabolism	**Glycolic acid**	COG0372	citrate synthase	K01647	CS, gltA	EC 2.3.1.1
		COG0065<br>COG1048	aconitate hydratase	K01681	ACO, acnA	EC 4.2.1.3
		COG1049	aconitate hydratase 2	K01682	gcnB	EC 4.2.1.3,<br>EC 4.2.1.99
		COG2224	isocitrate lyase	K01637	aceA	EC 4.1.3.1
		COG1052	Glycerate dehydrogenase	K00018	HprA	EC 1.1.1.29
		-	glyoxylate/hydroxypyruvate reductase	K00049	GRHPR	EC 1.1.1.79,<br>EC 1.1.1.81
		COG0111	glyoxylate/hydroxypyruvate reductase A	K12972	GhrA	EC 1.1.1.79,<br>EC 1.1.1.81
		-	glyoxylate/succinic semialdehyde reductase	K18121	GLYR	EC 1.1.1.79
Pyruvate metabolism	**lactic acid**	COG1304	L-lactate dehydrogenase	K00101	LldD	EC 1.1.2.3
	**Acetic acid**	COG2609	pyruvate dehydrogenase E1 component	K00163	Ace	EC 1.2.4.1
		COG1071	pyruvate dehydrogenase E1 component alpha subunit	K00161	PDHA, pdhA	EC 1.2.4.1
		COG0022	pyruvate dehydrogenase E1 component beta subunit	K00162	PDHB, pdhB	EC 1.2.4.1
		COG2609	pyruvate dehydrogenase E1 component	K00163	AceE	EC 1.2.4.1
		CoG0508	pyruvate dehydrogenase E2 component	K00627	DLAT, aceF, pdhC	EC 2.3.1.12
		COG0365	acetyl-CoA synthetase	K01895	ACSS, acs	EC 6.2.1.1
		-	acetaldehyde dehydrogenase	K00132		EC 1.2.1.10
		COG1012	acetaldehyde dehydrogenase / alcohol dehydrogenase	K04072	adhE	EC 1.2.1.10
		COG4569	acetaldehyde dehydrogenase	K04073	MhpF	EC 1.2.1.10
		COG4569	acetaldehyde/propanal dehydrogenase	K18366	bphJ, xylQ, nahO, tesF	EC 1.2.1.10,<br>EC 1.2.1.87
		COG1012	aldehyde dehydrogenase	K04021	EutE	EC 1.2.1.10
		COG1012	aldehyde dehydrogenase	K00128	ALDH	EC 1.2.1.3
		-	aldehyde dehydrogenase family 7 member A1	K14085	ALDH7A1	EC 1.2.1.31,<br>EC 1.2.1.8,<br>EC 1.2.1.3
		-	aldehyde dehydrogenase family 9 member A1	K00149	ALDH9A1	EC 1.2.1.47<br>EC 1.2.1.3
		COG1012	aldehyde dehydrogenase	K00138	AldB	EC 1.2.1.-

Citrate cycle (TCA cycle	**Citrate**	COG2609	pyruvate dehydrogenase E1 component	K00163	AceE	EC 1.2.4.1
		COG1071	pyruvate dehydrogenase E1 component alpha subunit	K00161	PDHA, pdhA	EC 1.2.4.1
		COG0022	pyruvate dehydrogenase E1 component beta subunit	K00162	PDHB, pdhB	EC 1.2.4.1
		COG0508	pyruvate dehydrogenase E2 component	K00627	DLAT, aceF, pdhC	EC 2.3.1.12
		COG0372	citrate synthase	K01647	CS, gltA	EC 2.3.3.1
	**succinate**	COG0372	citrate synthase	K01647	CS, gltA	EC 2.3.3.1
		-	malate dehydrogenase	K00025	MDH1	EC 1.1.1.37
		-	malate dehydrogenase	K00026	MDH2	EC 1.1.1.37
		COG0039	malate dehydrogenase	K00024	Mdh	EC 1.1.1.37
		COG0579	malate dehydrogenase (quinone)	K00116	Mqo	EC 1.1.5.4
		COG1838 COG1951	fumarate hydratase, class I	K01676	fumA, fumB	EC 4.2.1.2
		COG1951	fumarate hydratase subunit alphaalsD	K01677	fumA	EC 4.2.1.2
		COG1838	fumarate hydratase subunit beta	K01678	fumB	E4.2.1.2AB
		COG0114	fumarate hydratase, class II	K01679	fumC	E4.2.1.2B
		COG1053	Succinate dehydrogenase (ubiquinone) flavoprotein subunit	K00234	SDHA, SDH1	EC 1.3.5.1
		COG0479	Succinate dehydrogenase (ubiquinone) iron-sulfur subunit	K00235	SDHB, SDH2	EC 1.3.5.1
		COG2009	Succinate dehydrogenase (ubiquinone) cytochrome b560 subunit	K00236	SDHC, SDH3	-
		COG2142	Succinate dehydrogenase (ubiquinone) membrane anchor subunit	K00237	SDHD, SDH4	-
		COG1053	succinate dehydrogenase / fumarate reductase, flavoprotein subunit	K00239	sdhA, frdA	EC 1.3.5.1, EC 1.3.5.4
		COG0479	succinate dehydrogenase / fumarate reductase, iron-sulfur subunit	K00240	sdhB, frdB	EC 1.3.5.1, EC 1.3.5.4
		COG2009	succinate dehydrogenase / fumarate reductase, cytochrome b subunit	K00241	sdhC, frdC	-
		COG2142	succinate dehydrogenase / fumarate reductase, membrane anchor subunit	K00242	sdhD, frdD	-
		-	succinate dehydrogenase / fumarate reductase, subunit D	K18859	sdhD, frdD	-
		-	putative succinate dehydrogenase / fumarate reductase, subunit D	K18860	sdhD, frdD	-
		COG1053	fumarate reductase flavoprotein subunit	K00244	FrdA	EC 1.3.5.4
		COG0479	fumarate reductase iron-sulfur subunit	K00245	FrdB	EC 1.3.5.4
		COG3029	fumarate reductase subunit C	K00246	FrdC	-
		COG3080	fumarate reductase subunit D	K00247	frdD	-
		COG1053	succinate dehydrogenase / fumarate reductase, flavoprotein subunit	K00239	sdhA, frdA	EC 1.3.5.1, EC 1.3.5.4
		COG0479	succinate dehydrogenase / fumarate reductase, iron-sulfur subunit	K00240	sdhB, frdB	EC 1.3.5.1, EC 1.3.5.4
		COG2009	succinate dehydrogenase / fumarate reductase, cytochrome b subunit	K00241	sdhC, frdC	-
		COG2142	succinate dehydrogenase / fumarate reductase, membrane anchor subunit	K00242	sdhD, frdD	-
		-	succinate dehydrogenase / fumarate reductase, subunit D	K18859	sdhD, frdD	-
		-	putative succinate dehydrogenase / fumarate reductase, subunit D	K18860	sdhD, frdD	-

	**Phosphonate cluster (phn)**	COG3624	Alpha-D-ribose 1-methylphosphonate 5-triphosphate synthase subunit PhnG	K06166	PhnG	EC 2.7.8.37
		COG3627	Alpha-D-ribose 1-methylphosphonate 5-phosphate C-P lyase	K06163	Phnj	EC 4.7.1.1
		COG3625	Alpha-D-ribose 1-methylphosphonate 5-triphosphate synthase	K06165	PhnH	EC 2.7.8.37
		COG3626	Alpha-D-ribose 1-methylphosphonate 5-triphosphate synthase subunit PhnI	K06164	PhnI	EC 2.7.8.37
		COG0454	PhnO protein	K09994	PhnO	EC 2.3.1.-
		COG2764	PhnB protein	K04750	PhnB	-
		COG1524	phosphonoacetate hydrolase	K19670	PhnA	EC 3.11.1.2
		COG3709	ribose 1,5-bisphosphokinase	K05774	PhnN	EC 2.7.4.23
		COG1235	phosphoribosyl 1,2-cyclic phosphate phosphodiesterase	K06167	PhnP	EC 3.1.4.55
		COG4107	putative phosphonate transport system ATP-binding protein	K05781	PhnK	-
		COG4778	Alpha-D-ribose 1-methylphosphonate 5-triphosphate synthase subunit PhnL	K05780	PhnL	EC 2.7.8.37
		COG3639	phosphonate transport system permease protein	K02042	phnE	-
		COG2188	GntR family transcriptional regulator, phosphonate transport system regulatory protein	K02043	PhnF	-

Table S7 Gene annotations of *P. agglomerans* strain P5 genome related to its phosphate & Phosphonate transportation ability

	Trait	COG ID	Gene annotation	KO/Gene ID	KEGG GENE NAME	EC Number
**ABC transporters**	**Phosphonate transporter**	COG3639	phosphonate transport system permease protein	K02042	phnE2	-
		COG3221	phosphate-binding protein of phosphonate ABC transporter	K02044	phnD	-
		COG3638	phosphonate transport system ATP-binding protein	K02041	PhnC	EC 3.6.3.28
	**Phosphate transporter**	COG0226	phosphate transport system substrate-binding protein	K02040	Pst S	-
		COG0581	phosphate transport system permease protein	K02038	Pst A	-
		COG0573	phosphate transport system permease protein	K02037	Pst C	-
		COG1117	phosphate transport system ATP-binding protein	K02036	Pst B	EC 3.6.3.27

Table S8 Gene annotations of *P. agglomerans* strain *P5* genome related to its H2S production ability

	Trait	COG ID	Gene annotation	KO/Gene ID	KEGG GENE NAME	EC Number
**Sulfur metabolism**	**H2S production**	COG2897	thiosulfate/3-mercaptopyruvate sulfurtransferase	K01011	TST, MPST, sseA	EC 2.8.1.1
		COG0607	thiosulfate sulfurtransferase	K02439	glpE	EC 2.8.1.1
		COG0369	sulfite reductase (NADPH) flavoprotein alpha-component	K00380	cysJ	EC 1.8.1.2
		COG0155	sulfite reductase (NADPH) hemoprotein beta-component	K00381	cysI	EC 1.8.1.2
		-	3'-phosphoadenosine 5'-phosphosulfate synthase	K13811	PAPSS	EC 2.7.7.4<br>EC 2.7.1.25
		COG2895<br>COG0529	bifunctional enzyme CysN/CysC	K00955	cysNC	EC 2.7.7.4<br>EC 2.7.1.25
		COG2895	sulfate adenylyltransferase subunit 1	K00956	cysN	EC 2.7.7.4
		COG0175	sulfate adenylyltransferase subunit 2	K00957	cysD	EC 2.7.7.4
		COG0529	adenylylsulfate kinase	K00860	cysC	EC 2.7.1.25
		COG0175	phosphoadenosine phosphosulfate reductase	K00390	cysH	EC 1.8.4.8<br>EC 1.8.4.10
		COG0583	transcriptional regulator CysB	K13634	cysB	-
		COG2141	alkanesulfonate monooxygenase	K04091	ssuD	EC 1.14.14.5
		COG0431	FMN reductase	K00299	ssuE	EC 1.5.1.38
		COG2175	taurine dioxygenase	K03119	tauD	EC 1.14.11.17
		COG0626	cystathionine gamma-synthase	K01739	metB	EC 2.5.1.48
**Cysteine and methionine metabolism**		COG0031	cysteine synthase A	K01738	cysK	EC 2.5.1.47
		COG0031	cysteine synthase B	K12339	cysM	EC 2.5.1.47
		-	L-3-cyanoalanine synthase/ cysteine synthase	K13034	ATCYSC1	EC 2.5.1.47
		-	cysteine synthase / O-phosphoserine sulfhydrylase / cystathionine beta-synthase	K10150	cysO	EC 2.5.1.47<br>EC 2.5.1.65<br>EC 4.2.1.22
		COG2873	O-acetylhomoserine/O-acetylserine sulfhydrylase	K17069	MET17	EC 2.5.1.49<br>EC 2.5.1.47
		-	aspartate aminotransferase, cytoplasmic	K14454	GOT1	EC 2.6.1.1
		COG0436	aspartate aminotransferase	K00812	aspB	EC 2.6.1.1
		COG1448	aspartate aminotransferase	K00813	aspC	EC 2.6.1.1
		COG0436	aspartate aminotransferase	K11358	yhdR	EC 2.6.1.1
		COG2897	thiosulfate/3-mercaptopyruvate sulfurtransferase	K01011	TST, MPST, sseA	EC 2.8.1.1<br>EC 2.8.1.2

Table S9 Gene annotations of *P. agglomerans* strain P5 genome related to its H2S production ability

	Trait	COG ID	Gene annotation	KO/Gene ID	KEGG GENE NAME	EC Number
ABC transporters	**Sulfate transport system**	COG1613	sulfate transport system substrate-binding protein	K02048	cysP, sbp	-
		COG0555	sulfate transport system permease protein	K02046	cysU	-
		COG0555	sulfate transport system permease protein	K02047	cysW	-
		COG1118	sulfate transport system ATP-binding protein	K02045	cysA	EC 3.6.3.25
	**Cystine transport system**	COG0765	cystine transport system permease protein	-	ABC.CYST.P	-
		COG0834	cystine transport system substrate-binding protein	K02424	fliY	-
		COG1126	cystine transport system ATP-binding protein	K10010	ABC.CYST.A	EC 3.6.3.-
	**Taurine transport system**	COG4521	taurine transport system substrate-binding protein	K15551	tauA	-
		COG0600	taurine transport system permease protein	K15552	tauC	-
		COG4525	taurine transport system ATP-binding protein	K10831	tauB	EC 3.6.3.36
	**Sulfonate transport system**	COG0715	sulfonate transport system substrate-binding protein	K15553	ssuA	-
		COG0600	sulfonate transport system permease protein	K15554	ssuC	-
		COG1116	sulfonate transport system ATP-binding protein	K15555	ssuB	EC:3.6.3.-

Table S10 Gene annotations of *P agglomerans* strain P5 genome related to its secretion system

Bacterial secretion system	COG ID	Gene annotation	KO/Gene ID	KEGG GENE NAME	EC Number
**Type I**	COG1538	Outer membrane protein	K12340	tolC	-
**Type III**	-	type III secretion protein C	K03219	yscC, sctC	-
	-	type III secretion protein W	K04058	yscW, sctW	-
	COG1766	type III secretion protein J	K03222	yscJ, sctJ, hrcJ	-
	COG1338	type III secretion protein R	K03226	yscR, sctR, hrcR	-
	COG1987	type III secretion protein S	K03227	yscS, sctS, hrcS	-
	COG1684	type III secretion protein T	K03228	yscT, sctT, hrcT	-
	COG1377	type III secretion protein U	K03229	yscU, sctU, hrcU	-
	COG1298	type III secretion protein V	K03230	yscV, sctV, hrcV	-
	COG1157	ATP synthase in type III secretion protein N	K03224	yscN, sctN, hrcN	EC 3.6.3.14
	COG1886	type III secretion protein Q	K03225	yscQ, sctQ, hrcQ	-
	COG1317	type III secretion protein L	K03223	yscL, sctL	-
**Type sec-SRP**	COG0342	preprotein translocase subunit SecD	K03072	secD	-
	COG0690	preprotein translocase subunit SecE	K03073	secE	-
	COG1314	preprotein translocase subunit SecG	K03075	secG	-
	COG0201	preprotein translocase subunit SecY	K03076	secY	-
	COG1862	preprotein translocase subunit YajC	K03210	yajC	-
	COG0706	YidC/Oxa1 family membrane protein insertase	K03217	yidC,spoIIIJ,OXA1, ccfA	-

	COG0653	preprotein translocase subunit SecA	K03070	secA	-
	-	secretion monitor	K13301	secM	-
	COG0552	fused signal recognition particle receptor	K03110	ftsY	-
	COG1952	preprotein translocase subunit SecB	K03071	secB	-
	COG0541	signal recognition particle subunit SRP54	K03106	SRP54, ffh	EC 3.6.5.4
**Twin arginine targeting (Tat)**	COG1826	sec-independent protein translocase protein TatA	K03116	tatA	-
	COG1826	sec-independent protein translocase protein TatB	K03117	tatB	-
	COG0805	sec-independent protein translocase protein TatC	K03118	tatC	-
	COG1826	sec-independent protein translocase protein TatE	K03425	tatE	-
**Type VI**	COG3501	type VI secretion system secreted protein VgrG	K11904	vgrG	-
	COG3157	type VI secretion system secreted protein Hcp	K11903	Hcp	-
	COG3521	type VI secretion system protein VasD	K11906	vasD, lip	-
	COG3523	type VI secretion system protein ImpL	K11891	impL, vasK, icmF	-
	COG0542	type VI secretion system protein VasG	K11907	vasG, clpV	-
	COG3455	type VI secretion system protein ImpK	K11892	impK, ompA, vasF, dotU	-

Table S11 Gene annotations of *P. agglomerans* strain P5 genome related to its of phytohormones biogenesis ability.

	Trait	COG ID	Gene annotation	KO/Gene ID	KEGG GENE NAME	EC Number
**Tryptophan metabolism**	**IAA production**	COG0154	Amidase	K01426	amiE	EC 3.5.1.4
		COG0076	aromatic-L-amino-acid decarboxylase	K01593	DDC	EC 4.1.1.28
		COG1012	aldehyde dehydrogenase (NAD+)	K00128	ALDH	EC 1.2.1.3
		-	aldehyde dehydrogenase family 7 member A1	K14085	ALDH7A1	EC 1.2.1.31<br>EC 1.2.1.8<br>EC 1.2.1.3
		-	aldehyde dehydrogenase family 9 member A1	K00149	ALDH9A1	EC 1.2.1.47,<br>EC 1.2.1.3
**Butanoate pathway**	**3-hydroxy-2-butanone (acetoin)**	COG0028	acetolactate synthase I/II/III large subunit	K01652	ilvB, ilvG, ilvI	EC 2.2.1.6
		COG0440	acetolactate synthase I/III small subunit	K01653	ilvH, ilvN	EC 2.2.1.6
		COG3978	acetolactate synthase II small subunit	K11258	IlvM	EC 2.2.1.6
		COG3527	acetolactate decarboxylase	K01575	alsD, budA, aldC	EC 4.1.1.5
		COG1028	meso-butanediol dehydrogenase / (S, S)-butanediol dehydrogenase / diacetyl reductase	K03366	butA, budC	EC 1.1.1.-<br>EC 1.1.1.76<br>EC 1.1.1.304
		COG1028	meso-butanediol dehydrogenase / (S, S)-butanediol dehydrogenase / diacetyl reductase	K18009	budC	EC 1.1.1.-<br>EC 1.1.1.76<br>EC 1.1.1.304
		COG1028	meso-butanediol dehydrogenase / (S, S)-butanediol dehydrogenase / diacetyl reductase	K03366	butA, budC	EC 1.1.1.-<br>EC 1.1.1.76<br>EC 1.1.1.304
		COG1028	meso-butanediol dehydrogenase / (S, S)-butanediol dehydrogenase / diacetyl reductase	K18009	budC	EC 1.1.1.-<br>EC 1.1.1.76<br>EC 1.1.1.304
	**2,3-butanediol**	COG1063	(R, R)-butanediol dehydrogenase / meso-butanediol dehydrogenase / diacetyl reductase	K00004	BDH, butB	EC 1.1.1.4<br>EC 1.1.1.-<br>EC 1.1.1.303
		COG1028	meso-butanediol dehydrogenase / (S, S)-butanediol dehydrogenase / diacetyl reductase	K03366	butA, budC	EC 1.1.1.-<br>EC 1.1.1.76<br>EC 1.1.1.304

		COG0028	acetolactate synthase catalytic subunit	K01652	ilvB, ilvG, ilvI	EC 2.2.1.6L
		COG3978	acetolactate synthase II small subunit	K11258	ilvM	EC 2.2.1.6
		COG0440	acetolactate synthase I/III small subunit	K01653	ilvH, ilvN	EC 2.2.1.6S
		COG0028	acetolactate synthase I/II/III large subunit	K01652	ilvB, ilvG, ilvI	EC 2.2.1.6L
		COG0028	acetolactate synthase large subunit	K01576	MdlC	EC 2.2.1.6
		COG3527	alpha-acetolactate decarboxylase	K01575	alsD	EC 4.1.1.5
	**GABA**	COG1012	succinate-semialdehyde dehydrogenase/glutarate-semialdehyde dehydrogenase	K00135	gabD	EC 1.2.1.16<br>EC 1.2.1.79<br>EC 1.2.1.20
		COG1012	succinate-semialdehyde dehydrogenase	K08324	Sad	EC 1.2.1.16<br>EC 1.2.1.24
			succinate-semialdehyde dehydrogenase	K00139	ALDH5A1	EC 1.2.1.24
			succinate-semialdehyde dehydrogenase, mitochondrial	K17761	SSADH	EC 1.2.1.24
		COG0160	4-aminobutyrate aminotransferase / (S)-3-amino-2-methylpropionate transaminase	K13524	ABAT	EC 2.6.1.19<br>EC 2.6.1.22
		COG0160<br>COG4992	4-aminobutyrate aminotransferase / (S)-3-amino-2-methylpropionate transaminase	K07250	gabT	EC 2.6.1.19<br>EC 2.6.1.22
		COG0160<br>COG4992	5-aminovalerate/4-aminobutyrate aminotransferase]	K14268	davT, gabT	EC:2.6.1.48<br>EC 2.6.1.19
		COG0160<br>COG4992	4-aminobutyrate aminotransferase	K00823	puuE	EC 2.6.1.19
	**4-hydroxybenzoate Production**	COG3161	chorismate-pyruvate lyase	-	ubiC	EC 4.1.3.40

Table S12 Gene annotations of *P agglomerans* strain P5 genome related to its siderophore biogenesis ability

	Trait	COG ID	Gene annotation	KO/Gene ID	KEGG GENE NAME	EC Number
**Biosynthesis of siderophore group nonribosomal peptides**	**Siderophore**	COG1535<br>COG3433	bifunctional isochorismate lyase / aryl carrier protein	K01252	entB, dhbB, vibB, mxcF	EC 3.3.2.1,<br>EC 6.3.2.14
		COG1021	2,3-dihydroxybenzoate-AMP ligase	K02363	entE, dhbE, vibE, mxcE	EC 6.3.2.14,<br>EC 2.7.7.58
		COG1020<br>COG3319	enterobactin synthetase component F	K02364	entF	EC 6.3.2.14
		COG0477	Enterobactin exporter	K08225	EntS	-
		COG1028	2,3-dihydro-2,3-dihydroxybenzoate dehydrogenase	K00216	entA	EC 1.3.1.28
		COG1169	salicylate biosynthesis isochorismate synthase	K01851	pchA	EC 5.4.4.2
		COG1169	isochorismate synthase	K02361	entC	EC 5.4.4.2
		COG1169	menaquinone-specific isochorismate synthase	K02552	menF	EC 5.4.4.2
		COG0147	salicylate synthetase	K04781	mbtI, irp9, ybtS	EC 5.4.4.2,<br>EC 4.2.99.21
		COG0614	Ferric enterobactin-binding periplasmic protein	K02016	FepB	-

	COG0609	Ferric enterobactin transport system permease protein	K02015	FepD	-
	-	Ferric enterobactin transport system permease protein	-	FepG	-
		Ferric enterobactin transport ATP-binding protein	-	FepC	-
	COG2382	Enterobactin esterase	K07214	Fes	-
	-	TonB-dependent receptor; Outer membrane receptor for ferric enterobactin and colicins B, D	K19611	GAPWK	-
	-	Putative OMR family iron-siderophore receptor precursor"	-	-	-
